# Vaccination Protocols in Canine Shelters in Northern Italy: A Survey of Current Practices

**DOI:** 10.3390/ani16142276

**Published:** 2026-07-22

**Authors:** Mara Battilani, Alberto Provenzano, Andrea Balboni, Lorenzo Scagliarini

**Affiliations:** 1 Department of Veterinary Medical Sciences, Alma Mater Studiorum-University of Bologna, 40064 Ozzano Emilia, Italy; provenzanoalberto@gmail.com (A.P.); a.balboni@unibo.it (A.B.); 2 Veterinary Public Health Unit, Azienda USL di Bologna (AUSL Bologna), 40121 Bologna, Italy; lorenzo.scagliarini@ausl.bologna.it

**Keywords:** canine shelters, vaccination protocols, WSAVA vaccination guidelines, shelter medicine

## Abstract

Vaccination is a fundamental tool for the prevention and control of infectious diseases in canine shelters, where high population density and frequent animal turnover increase the risk of pathogen transmission. However, in Italy there is currently no unified regulatory framework governing vaccination protocols for shelter dogs. In this context, the aim of this study was to evaluate vaccination protocols and preventive health practices adopted in canine shelters in the province of Bologna (Northern Italy). Data were collected through on-site visits and review of institutional documentation and collection of information from shelter personnel using a structured data collection instrument designed to gather information on vaccination protocols, vaccine types, administration intervals, epidemiological history of the facilities, and preventive measures. Additionally, vaccination records from a sample of dogs in each shelter were examined to compare the protocols formally adopted with the vaccinations actually administered. The analysis revealed substantial heterogeneity among shelters in the vaccination protocols applied, largely reflecting the professional discretion of veterinary practitioners. Furthermore, several inconsistencies and errors in vaccination schedules were identified, which may compromise immunological protection within the shelter population. These findings underline the importance of implementing standardized vaccination protocols tailored to the local epidemiological context to improve disease prevention and health management in canine shelters.

## Simple Summary

Vaccination plays a crucial role in preventing and controlling infectious diseases in canine shelters, where dogs are housed in close proximity, and the risk of disease spread is high. Despite its importance, Italy lacks a unified regulatory framework defining vaccination protocols for shelter dogs. This study investigated vaccination practices in ten canine shelters located in the province of Bologna (Northern Italy). Information was collected through on-site visits and review of institutional documentation and collection of information from shelter personnel, using a structured data collection instrument to record vaccination protocols, vaccine types, administration intervals, epidemiological history, and preventive health measures. Vaccination records from a sample of dogs in each facility were also examined to verify whether the protocols formally adopted corresponded to the vaccinations actually administered. The results revealed marked variability among shelters in vaccination strategies, mainly reflecting the discretionary decisions of the veterinary practitioners responsible for each facility. In several cases, inconsistencies and errors in vaccination schedules were identified, potentially compromising the level of protection achieved. These findings highlight the need for more standardized vaccination strategies based on local epidemiological risk in order to improve disease prevention and health management in shelter dog populations.

## 1. Introduction

Vaccination is a key strategy for the prevention and control of infectious diseases associated with high morbidity and mortality in both human and animal populations [1]. Vaccines are designed to induce a specific and protective immune response against pathogens, ideally providing long-lasting immunity and reducing disease transmission [2]. In veterinary medicine, vaccination plays a central role not only in safeguarding animal health and welfare but also in reducing the need for antimicrobial treatments, thereby contributing to the mitigation of antimicrobial resistance (AMR) [3].

Within a One Health framework, vaccination of animal populations is also crucial for limiting zoonotic disease transmission. It is estimated that approximately 60% of human infectious diseases originate from animals, highlighting the importance of integrated prevention strategies [4,5]. In this context, effective vaccination programs in domestic animals can significantly reduce the risk of pathogen transmission to humans.

Vaccination guidelines for companion animals have been developed by several international organizations, including the World Small Animal Veterinary Association (WSAVA), which periodically updates evidence-based recommendations for dogs and cats [6,7,8]. These guidelines classify vaccines as core, non-core, or not recommended and emphasize the importance of tailoring vaccination protocols to the individual animal’s lifestyle and to local epidemiological conditions. Core vaccines are considered essential for all animals due to their protection against severe and globally distributed diseases, whereas non-core vaccines are recommended based on specific risk factors such as lifestyle and geographic location [6,7]. In dogs, core vaccines typically include those against canine distemper virus (CDV), canine parvovirus (CPV), and canine adenovirus (CAV), whereas vaccines against respiratory pathogens such as canine parainfluenza virus (CPiV) and *Bordetella bronchiseptica* are generally classified as non-core, as their use depends on the epidemiological context and individual risk factors [9].

In addition, some vaccines classified as non-core may be promoted to core and recommended for the entire canine population in regions where the disease is endemic. In the Italian context, leptospirosis vaccination represents a paradigmatic example of this situation, as it is an endemic, life-threatening, and zoonotic disease whose indication is strongly influenced by local epidemiological conditions and exposure risk [6]. Recent investigations conducted in shelter dogs in Northern Italy have reported serological positivity rates exceeding 40% and documented the circulation of several pathogenic serogroups, including Icterohaemorrhagiae, Copenhageni, Grippotyphosa, Bratislava, Canicola, and Pomona. Importantly, most of the commonly detected serogroups are included in currently available multivalent vaccines, supporting the relevance of vaccination as a preventive measure in high-risk environments such as shelters [10].

These findings support current recommendations that, in regions where canine leptospirosis is endemic and appropriate vaccines are available, vaccination of all dogs should be strongly encouraged and may be considered a core component of preventive healthcare protocols [6,11]. Particular attention is required in high-density environments like animal shelters, where dogs are exposed to an increased risk of infectious disease transmission. Factors such as overcrowding, high animal turnover, stress, and frequent contact with unfamiliar individuals contribute to a higher incidence of infectious diseases compared with privately owned pets [12,13]. For this reason, vaccination represents a cornerstone of disease prevention in shelter medicine, often requiring adapted protocols that ensure rapid onset of immunity and adequate population coverage [14]. Vaccination strategies in shelter environments therefore require a flexible, risk-based approach that integrates general recommendations with site-specific epidemiological factors [6,12,15]. Accordingly, in shelter settings, vaccines against *B. bronchiseptica* and CPiV, although generally classified as non-core for privately owned dogs, should also be considered core because of the high risk of exposure, rapid transmission, and widespread morbidity associated with respiratory disease outbreaks [6,9].

Modified-live vaccines are generally preferred in shelter settings due to their faster onset of immunity and ability to overcome maternal antibody interference more effectively than inactivated vaccines [6,7,12]. Early and appropriately scheduled vaccination is therefore critical, particularly for young animals entering the shelter environment [15,16].

Despite the availability of international guidelines, vaccination practices may vary considerably depending on local conditions and professional judgment. In Italy, there is currently no unified regulatory framework defining standardized vaccination protocols for dogs housed in shelters. Furthermore, the implementation of “no-kill” policies has contributed to the expansion of long-term shelter populations, often resulting in overcrowded facilities and increased epidemiological risk [17].

Italian legislation distinguishes between different types of canine facilities, including sanitary shelters (“canili sanitari”) and long-term shelters (“canili rifugio”) [17], each with specific roles in the management of stray dog populations. Sanitary kennels are facilities intended for the temporary housing and quarantine of newly captured stray dogs, whereas long-term shelters accommodate dogs that have not been reclaimed by their owners or adopted following the quarantine period, as well as dogs relinquished by their owners. According to 281/91 Italian law, stray dogs found in the territory are captured and initially transferred to sanitary shelters, where they undergo a mandatory quarantine period (minimum 10 days). After this period, if they are not returned to their owners or adopted, they are transferred to long-term shelters, where they will remain until adoption or death. In the Emilia-Romagna region, however, all canine shelters simultaneously perform both sanitary kennel and long-term shelter functions within the same facility. However, dedicated sanitary units are maintained for quarantine and health monitoring of newly admitted dogs, and these areas are structurally and functionally separated from the long-term housing units. Following the quarantine period, dogs that are not reclaimed or adopted are transferred to the shelter section, where they remain until adoption or death.

However, limited information is available regarding how vaccination protocols are actually implemented in these settings and whether they are applied consistently.

In this context, the aim of this study was to evaluate vaccination protocols and preventive health practices in canine shelters in the province of Bologna (Emilia-Romagna region, Northern Italy), with particular focus on their adherence to current vaccination guidelines.

## 2. Materials and Methods

### 2.1. Study Population and Shelter Selection

This cross-sectional observational study was conducted in canine shelters located in the province of Bologna (Emilia-Romagna region, Northern Italy), under the supervision of the Local Health Authority (AUSL Bologna).

Shelter selection was conducted in collaboration with the Local Health Authority (AUSL Bologna) and considered all canine shelters under the veterinary oversight of AUSL Bologna within the province of Bologna. At the time of the study, 13 canine shelters were present in the province. Of these, 10 were included in the study and visited between October 2023 and April 2024; two facilities were excluded because no dogs were housed during the data collection period, whereas one shelter, despite being geographically located within the province of Bologna, was under the jurisdiction of a different Local Health Authority. Most participating facilities were municipal or intermunicipal shelters, while only one was a privately managed shelter. Since the study aimed to provide a descriptive overview of vaccination practices in the province of Bologna, all eligible shelters under the veterinary oversight of AUSL Bologna were enrolled.

According to Emilia-Romagna Regional Law No. 27/2000 [18], canine shelters in the Emilia-Romagna region simultaneously perform both sanitary kennel (“canili sanitari”) and long-term shelter (“canili rifugio”) functions within the same facility. Nevertheless, although both functions coexist within the same shelter structure, quarantine and sanitary isolation units are structurally and functionally separated from areas dedicated to the long-term housing of resident dogs.

The shelters included in the study showed marked variability in capacity, population, size, and management complexity. Information regarding the epidemiological history of each facility, including outbreaks of vaccine-preventable infectious diseases during the previous five years, was also collected.

This study was observational in nature, and no animals were subjected to experimental procedures, invasive sampling, or additional diagnostic testing specifically for research purposes.

### 2.2. Data Collection and Checklist Design

A structured data collection instrument was specifically developed to standardize the collection of information regarding vaccination protocols and preventive healthcare practices implemented in the shelters (Table 1).

Data collection was performed through on-site visits and structured collection of information from shelter managers and veterinary directors responsible for the health management of each facility, in the presence of a veterinarian from the Local Health Authority (AUSL Bologna). The data collection instrument included thematic sections covering:

(i) General shelter information;
(ii) Health management procedures for newly admitted animals;
(iii) Infectious disease events reported during the previous five years;
(iv) Vaccination protocols for puppies, adult dogs, and newly admitted dogs;
(v) Vaccination schedules and revaccination intervals;
(vi) Vaccine formulations used;
(vii) Classification of shelters according to the Regional Control Plan for Canine Leishmaniasis [19], which categorizes facilities into four risk categories based on the presence of sandfly vectors and/or seropositive dogs, each associated with specific surveillance, diagnostic, and control measures;
(viii) Preventive healthcare measures, including ecto- and endoparasite prophylaxis, disinfection procedures, disinfestation programs, and rodent control measures.

Vaccination records and vaccination booklets were examined on a sampling basis to verify the actual implementation of the declared vaccination protocols. Vaccination records consisted primarily of individual paper vaccination booklets routinely used in veterinary practice, containing information on vaccine administration dates, vaccine identification labels, and veterinarian certification. These records were reviewed to verify consistency between the vaccination protocols declared by each shelter and their practical implementation. The primary source of information regarding vaccination practices consisted of the official health management protocols adopted by each shelter. Vaccination booklets were reviewed on a sampling basis as an audit procedure to verify consistency between declared and implemented practices. At the time of the visits, only a limited number of vaccination booklets were available for review in some facilities, particularly for puppies or recently admitted dogs that had already been adopted or transferred. Since complete vaccination records for all housed dogs were not systematically available, a limited number of records (generally 2–3 dogs per shelter) were examined in each facility to evaluate the consistency between declared and applied vaccination protocols.

Additional information was collected regarding clinical management procedures for newly admitted dogs, including quarantine measures, timing of clinical examination, diagnostic screening procedures, antiparasitic prophylaxis, and timing of vaccination after admission. Although not the primary focus of this study, information on parasite control and sanitation measures was collected to provide a broader overview of health management practices within the shelters.

**Table 1:** Structured data collection instrument used in canine shelters.

Section	Checklist Items
General information	Canine shelter name and location
Health management	Health protocol for newly admitted animals
Infectious diseases history	Infectious disease events reported in the previous 5 years
Vaccination protocols	Puppies
	Adult dogs
	Newly admitted adult dogs
Vaccination schedules	Age at first vaccination
	Booster intervals
	Revaccination frequency
Vaccines and risk classification	Type of vaccines used (vaccine formulations)
	Risk classification according to the Regional Canine Leishmaniasis Surveillance Program
Preventive measures	Ecto- and endoparasite prophylaxis
	Disinfection, disinfestation, and rodent control procedures

## 3. Results

The ten shelters included in the study systematically vaccinated dogs against core diseases (canine parvovirosis, distemper, and infectious hepatitis), canine parainfluenza virus, and leptospirosis, despite the absence of a mandatory national vaccination framework [6,12]. Data on infectious disease history (outbreaks in the last five years), vaccination protocols, and vaccination schedules are summarized in Table 2.

A total of seven episodes of infectious disease were reported across the shelters over the past five years. These included three reported parvovirosis events in shelters n. 1, n. 4, and n. 9; three leptospirosis events in shelters n. 1, n. 2, and n. 3; and one canine infectious respiratory disease complex (CIRDC) event in shelter n. 8. Several reported infectious disease events involved unvaccinated animals, particularly puppies originating from outside the shelters. Three events were associated with newly introduced puppies. Overall, in four out of seven reported events, the affected animals were either unvaccinated or had not completed the vaccination protocol, whereas vaccination status was unclear in the remaining two events.

Marked variability was observed in vaccination protocols for puppies. Only two shelters applied a structured vaccination schedule, including administrations at 8, 12, and 16 weeks of age for core vaccines and at 8 and 12 weeks for leptospirosis (shelters n. 1 and n. 9). Other shelters adopted simplified or incomplete protocols, with variability in the age at first vaccination, in the age of completion of the juvenile vaccination series, and by omission of vaccines recommended for high-risk shelter environments. In some cases, the starting age of vaccination was not specified, while one shelter did not provide a defined vaccination protocol (shelter n. 6).

For newly admitted dogs, all shelters reported vaccination after clinical evaluation, generally within 7–10 days from entry. One shelter vaccinated within 24 h (shelter n. 8), while another did not provide a clearly defined vaccination protocol for newly admitted dogs (shelter n. 10). Most shelters declared the administration of a booster dose 21–30 days after primary vaccination. However, the review of vaccination records showed that booster doses were not consistently administered in practice.

Inconsistencies were also observed in leptospirosis vaccination. In several cases, the recommended booster dose 3–4 weeks after primary vaccination was not administered. Additionally, discrepancies in vaccine formulation use were reported (e.g., switching between L2, L3, and L4 without appropriate booster protocols).

Adult dogs were vaccinated at different intervals depending on the shelter. Most shelters (6/10) performed annual revaccination for both core vaccines and leptospirosis, while others adopted longer intervals (3–4 years) for core vaccines, maintaining annual vaccination for leptospirosis.

None of the shelters used serological testing to assess antibody titers for vaccination management.

Data on parasite control and sanitation measures applied in shelters are reported in Table 3.

All shelters implemented disinfection protocols, although the frequency varied (Table 3). Only two shelters reported daily use of disinfectants (shelters n. 7 and n. 10), whereas the remaining shelters reported occasional use without a predefined application schedule. Regarding the Regional Control Plan for Canine Leishmaniasis [19], shelters included in the study were classified as categories 1 or 3 (Table 3). No shelters performed vaccination against *Leishmania infantum*, relying instead on preventive measures such as insecticidal collars, spot-on formulations, and vector control strategies. Rodent control measures were implemented in all shelters, typically through structured plans managed by external companies, with the exception of one facility (shelter n. 10), where control was managed internally.

## 4. Discussion

In high-risk environments such as canine shelters, vaccination represents a cornerstone for the prevention and control of infectious diseases. Vaccination strategies in shelter environments require a flexible, risk-based approach that integrates general recommendations with site-specific epidemiological factors [6,14,15].

Despite the recognized importance of vaccination in these settings, limited information is available on how vaccination protocols are actually implemented in practice, especially in the Italian context. Therefore, this study provides a field-based evaluation of vaccination protocols and preventive health practices in canine shelters in the province of Bologna (Emilia-Romagna region, Northern Italy), integrating data on infectious disease history, vaccination strategies, and biosecurity measures collected through a structured checklist.

The findings highlight that, despite the absence of a unified regulatory framework in Italy, all shelters included in the analysis systematically implement vaccination protocols, confirming widespread recognition among veterinary professionals regarding the importance of vaccination in high-risk environments such as shelters. However, important deviations from current evidence-based vaccination recommendations were identified in the practical implementation of these protocols [13,15]. The relatively low number of reported infectious disease events over the past five years may reflect the protective effect of vaccination strategies. However, the occurrence of infectious disease events, particularly among unvaccinated animals and newly introduced puppies, highlights the importance of timely vaccination and appropriate management of newly admitted animals as critical components of disease prevention. The role of incoming animals as a source of infection is well recognized in shelter medicine and is consistent with previous studies and guidelines [6,15]. Canine parvovirosis represented the most frequently reported infectious disease among the shelters examined in the present study, consistent with findings from a previous survey conducted in canine shelters in Portugal [20]. Although canine parvovirosis is a vaccine-preventable disease and vaccination against CPV has been routinely performed for decades, outbreaks still frequently occur in shelter environments. The persistence of CPV in canine communities is favored both by the high environmental resistance of the virus and by maternal antibody interference, which may compromise the effectiveness of early vaccinations in puppies [6,21]. In the present study, parvovirosis outbreaks were consistently associated with the introduction of unvaccinated puppies into the shelters, emphasizing once again the importance of appropriate vaccination protocols and early immunization in high-risk environments.

A key finding of this study is the marked heterogeneity in vaccination protocols across shelters, particularly for puppies. None of the facilities fully adhered to WSAVA recommendations, which suggest repeated vaccinations every 2–3 weeks from one month of age until at least 20 weeks, to overcome maternal antibody interference [6]. The lack of adherence to WSAVA guidelines may be partly explained by limited financial resources and operational constraints in shelters. Since the approval of Italian Law No. 281/1991, which prohibits the euthanasia of stray dogs, many animals entering shelters remain there for the rest of their lives. This has created a significant economic burden, as shelter management costs are high while funding dedicated to the control of stray dog populations remains limited despite the continued widespread presence of stray dogs in Italy, particularly in southern regions [22]. As a consequence, simplified or incomplete vaccination protocols are often adopted, which may result in insufficient immunological protection, particularly in high-risk environments. However, inadequate vaccination strategies may themselves generate substantial costs, as the management of infectious disease events often requires isolation measures, diagnostic investigations, treatments, enhanced biosecurity procedures, and additional personnel resources. Consequently, investment in appropriate vaccination protocols may represent a cost-effective preventive strategy in shelter environments.

Similarly, vaccination practices for newly admitted animals were not fully aligned with international guidelines, which recommend vaccination at or before entry into the shelter [12]. In all facilities included in this study, vaccination was performed after a delay following clinical evaluation, potentially increasing the window of susceptibility to infectious diseases. However, this aspect should be interpreted in light of the organizational structure of the shelters examined. Newly admitted dogs were initially housed in quarantine and sanitary isolation units that were structurally and functionally separated from long-term housing areas, generally in individual boxes without direct contact with resident dogs. Therefore, although delayed vaccination may increase susceptibility to infection, the actual risk of disease transmission is also strongly influenced by the effectiveness of biosecurity measures adopted within quarantine areas, including the use of personal protective equipment (PPE), hand hygiene, footwear sanitation, appropriate disinfection procedures, and controlled personnel movement between quarantine and permanent housing units. It should also be recognized that the risk of disease transmission is not limited to contact between newly admitted and resident dogs. Transmission may also occur among dogs housed within quarantine units, further emphasizing the importance of strict biosecurity measures.

In addition, the timing of vaccination reported by shelters may not always fully reflect routine clinical practice. In many facilities, veterinary practitioners are not permanently present on site and perform scheduled visits according to contractual agreements. Consequently, newly admitted dogs are often clinically evaluated within the first days after admission, and vaccination may be postponed until a subsequent veterinary visit following clinical assessment and, when necessary, antiparasitic treatment or diagnostic screening procedures. As a result, the interval between admission and vaccination may be longer than theoretically recommended by international guidelines.

Core vaccines for dogs in shelter environments include modified-live CDV, CPV, CAV, leptospirosis, CPiV, and *Bordetella bronchiseptica* [6,23]. None of the shelters included rabies vaccination in their protocols, since rabies vaccination is not mandatory in Italy, which has been officially recognized as rabies-free since 2013 [24]. All dogs housed in the shelters included in this study were vaccinated with modified-live vaccines against CDV, CPV, CAV, and CPiV. Vaccination against respiratory pathogens such as CPiV and *Bordetella bronchiseptica* represents an important component of disease prevention strategies in high-density environments such as shelters [12,25]. Although these vaccines do not confer sterilizing immunity, they may reduce the severity and frequency of clinical manifestations associated with canine infectious respiratory disease complex (CIRDC) [26]. Vaccination against CPiV was routinely performed in all shelters examined, whereas vaccination against *Bordetella bronchiseptica* was not adopted in any of the shelters included in this study, despite the WSAVA guidelines recommending administration of an intranasal modified-live *B. bronchiseptica* vaccine to all adult dogs and puppies at least 3 weeks of age upon admission to the shelter [6].

Vaccination against canine parainfluenza virus, due to its incomplete protective efficacy compared to other core vaccines, should be administered annually [6,7]. In the present study, only a limited number of shelters implemented annual vaccination against canine parainfluenza. In those shelters where annual revaccination was not performed, vaccine effectiveness is likely to be significantly reduced. This practice may be explained by the fact that adherence to triennial revaccination schedules for core vaccines would ideally require the use of single-antigen parainfluenza vaccines. However, in shelter settings, parainfluenza vaccination is commonly administered together with core vaccines due to practical and economic constraints.

The occurrence of a CIRDC event in shelter n. 8 despite annual vaccination against the parainfluenza virus is not unexpected, considering the multifactorial nature of this syndrome and the absence of vaccination against *Bordetella bronchiseptica* in all the shelters included in the study. CIRDC involves multiple viral and bacterial pathogens, including *Bordetella bronchiseptica*, canine adenovirus, and others [27]. Vaccination against CPiV alone may therefore be sufficient to prevent respiratory disease events in shelter environments. Furthermore, previous studies have shown that parenteral CPiV vaccines may provide incomplete protection against infection and viral shedding, whereas intranasal vaccination may offer superior local mucosal immunity [28]. This finding further underlines that vaccination alone may not be sufficient to prevent respiratory disease events in shelter environments, where additional factors such as stress, overcrowding, and ventilation play a crucial role [13].

Leptospirosis is widely recognized as an endemic zoonotic disease in many European countries, including Italy, and represents a significant concern for both animal and public health [29,30]. Although Leptospira vaccines are generally classified as non-core in international guidelines, their use is strongly recommended in endemic areas based on local epidemiological risk [6,11].

In this study, leptospirosis vaccination was widely implemented across shelters; however, several critical issues were identified. First, inconsistencies in the administration of booster doses were frequently observed. Given that leptospira vaccines are inactivated, the booster dose administered 3–4 weeks after primary vaccination is essential to ensure an adequate immunological response. Failure to perform this booster may result in incomplete or insufficient protection.

Second, the inappropriate use or switching of vaccine formulations (e.g., L2, L3, and L4) without correct revaccination protocols potentially affects immunological coverage. In particular, the use of tetravalent (L4) vaccines is generally recommended in endemic areas, as they provide broader protection against circulating serogroups compared to bivalent formulations [31]. However, when switching from formulations covering fewer serogroups (e.g., L2 or L3) to L4, an additional booster is required to ensure effective immunization against newly introduced antigens [32]. These findings highlight the importance of correct vaccine administration protocols in ensuring effective protection against leptospirosis.

The occurrence of a leptospirosis outbreak caused by serovar Sejroe, which is not included in commonly used L4 vaccines, is particularly relevant [33]. This finding confirms that currently available vaccines do not provide complete protection against all circulating serovars and highlights the dynamic and region-specific nature of leptospiral epidemiology [30]. Consequently, vaccination strategies should be integrated with epidemiological surveillance to better match vaccine coverage with circulating strains. It also reinforces the importance of combining vaccination strategies with environmental management and rodent control measures.

The consistent implementation of rodent control measures across all shelters is particularly relevant given the role of rodents as maintenance hosts for *Leptospira* spp. [34].

The variability observed in revaccination intervals for adult dogs reflects partial alignment with current evidence-based recommendations. While some shelters adopted extended intervals for core vaccines, others continued annual revaccination. The concurrent use of multivalent vaccines, including parainfluenza virus, which has a shorter duration of immunity, may partly explain this practice. The widespread use of annual revaccination protocols observed in this study appears to contrast with the current concept of extended duration of immunity (DOI) for core vaccines, which supports less frequent booster administration in low-risk populations [35,36] However, in high-risk environments such as shelters, characterized by high population turnover and continuous exposure to infectious agents, more frequent vaccination may still be considered a pragmatic approach to ensure adequate population immunity [6]. These findings highlight the need to balance evidence-based vaccination principles with the specific epidemiological context of shelter environments.

In small animal practice, serological testing may represent a valuable tool to support vaccination-related decision-making. The commercial availability of rapid in-practice test kits for the detection of antibodies against CDV, CPV, and CAV has increased the feasibility of antibody testing in routine veterinary medicine [37,38]. The absence of serological testing in all shelters represents a missed opportunity to optimize vaccination strategies, as recommended by WSAVA guidelines [6]. Although practical and economic constraints may limit its routine use, antibody titration could contribute to more rational revaccination protocols, improve vaccination in newly admitted dogs [39], and support outbreak control through targeted emergency vaccination [6].

From a One Health perspective, the consistent implementation of rodent control programs is particularly relevant, considering the zoonotic nature of leptospirosis and the role of rodents as maintenance hosts. Similarly, the reliance on vector control rather than vaccination for *Leishmania infantum* reflects current practices in endemic areas, where preventive measures are primarily focused on reducing vector exposure.

Finally, this study highlights that vaccination protocols are largely dependent on the individual clinical judgment of the responsible veterinarians. This may lead to variability in practices, particularly when personnel changes occur, potentially affecting the overall level of protection within shelters.

This study has some limitations, including the limited number of shelters included and the evaluation of vaccination records based on a small sample per facility, which may not fully represent all practices within each shelter. Although the shelters included in this study represent the majority of facilities operating under the supervision of AUSL Bologna, caution should be exercised when extrapolating these findings to shelters in other Italian regions, where management practices, available resources, and local epidemiological conditions may differ.

However, vaccination practices were primarily assessed through the analysis of official health management protocols and information provided by veterinary personnel. Therefore, the review of individual vaccination records should be regarded as an audit procedure aimed at verifying consistency between declared and implemented protocols rather than as a formal estimate of compliance rates within each shelter population.

Nevertheless, to the best of our knowledge, no previous studies have comprehensively evaluated vaccination practices in Italian canine shelters. In contrast to previous European surveys based mainly on questionnaire responses [20], the present study included direct on-site inspections, structured interviews, and examination of vaccination records, providing a more realistic and comprehensive overview of vaccination practices and preventive health management in shelter environments.

Overall, these findings highlight the gap between theoretical vaccination guidelines and their real-world implementation in shelter settings and emphasize the need for more standardized, evidence-based vaccination protocols that are aligned with international guidelines while remaining adaptable to local epidemiological conditions.

In particular, for diseases such as leptospirosis, vaccination strategies should be tailored to the specific epidemiological context, taking into account both pathogen circulation and the limitations of available vaccines.

Nevertheless, several positive aspects emerged from the present study. Despite the variability of vaccination protocols and the incomplete adherence to international guidelines, all shelters ensured vaccination of housed dogs against the main canine infectious diseases. In addition, rodent control programs, routine disinfection procedures, and antiparasitic treatments were consistently implemented across all facilities, reflecting a general commitment to infectious disease prevention and animal health management.

## 5. Conclusions

Vaccination represents a fundamental tool for the prevention and control of infectious diseases, particularly in high-density canine populations such as shelters, where effective immunization strategies are essential to ensure both individual animal welfare and overall population health. Despite the availability of international recommendations, this study highlights considerable variability in vaccination practices among shelters in the province of Bologna (Northern Italy), reflecting the absence of national or regional regulatory requirements for canine vaccination in shelter settings.

Several inconsistencies were identified in vaccination schedules, revaccination intervals, and the use of vaccines against leptospirosis and respiratory pathogens, potentially compromising the level of protection achieved. In many cases, vaccination strategies appeared to rely primarily on the individual clinical judgment of the veterinary directors rather than on structured, evidence-based protocols specifically adapted to local epidemiological conditions and shelter-related risk factors. These findings underline the need for clearer and more standardized vaccination protocols that remain sufficiently flexible to account for the operational and epidemiological characteristics of each facility.

Nevertheless, despite the heterogeneity of vaccination protocols and the financial constraints that frequently affect shelter management, all facilities included in this study ensured routine vaccination against the principal canine infectious diseases. This finding is particularly relevant considering that many shelters partially rely on external funding or donations for animal care and health management. In addition, all shelters implemented rodent control programs, disinfection procedures, and antiparasitic prophylaxis measures, demonstrating a substantial commitment to infectious disease prevention and animal health protection.

Furthermore, all shelters included in the study were equipped with dedicated quarantine and sanitary isolation areas for newly admitted or infectious animals, structurally and functionally separated from long-term housing units, in accordance with current regional regulations. These organizational and biosecurity measures likely represent an important additional factor contributing to infectious disease prevention and containment within shelter environments.

## Figures and Tables

**Table 2:** Vaccination protocols and schedules applied in canine shelters.

Shelter	Infectious Disease Events (Last 5 Years)	Puppies (≤16 Weeks)	Adult Dogs	Newly Admitted Dogs
1	Parvovirosis (2020, unvaccinated puppy); Leptospirosis (2020, unvaccinated adult)	CPV + CDV + CAV + CPiV (MLV): 8–12–16 weeks; L4: 8–12 weeks	CPV + CDV + CAV + CPiV (MLV) every 3 years; L4: annually	CPV + CDV + CAV + CPiV (MLV)+ L4: two doses 21–30 days apart
2	Leptospirosis (2020)	CPV + CDV 4 weeks CPV + CDV + CAV + L4: 8–11 weeks	CPV + CDV + CAV + CPiV + L4 annually	CPV + CDV + CAV + CPiV (MLVl) + L4 two doses 3 weeks apart
3	Leptospirosis (2021, ser. Sejroe)	CPV + CDV + CAV + CPiV (MLV) +L4 two doses 3–4 weeks apart; start age not defined	CPV + CDV + CAV + CPiV (MLV) +L4 annually	CPV + CDV + CAV + CPiV (MLV,) + L4 two doses 3 to 4 weeks apart
4	Parvovirosis (2022, imported litter)	CPV + CDV + CAV + CPiV (MLV), two doses 3–4 weeks apart; start age not defined	CPV + CDV + CAV + CPiV (MLV) every 4 years; L4 annually	CPV + CDV + CAV + CPiV (MLV) + L4 two doses 3–4 weeks apart
5	None	CPV + CDV + CAV + CPiV (MLV) +L4 two doses 21 days apart; start age not defined	CPV + CDV + CAV + CPiV (MLV) + L4 annually	CPV + CDV + CAV + CPiV (MLV) + L4 Not specified whether one or two doses; no defined vaccination protocol
6	None	^§^ Not specified	CPV + CDV + CAV + CPiV Every 2–4 years L4 annually L2 every 6 months	^§^ Not specified
7	None	CPV + CDV + CAV + CPiV + L4 at ~60 days + booster after 21 days	CPV + CDV + CAV + CPiV + L4 annually	CPV + CDV + CAV + CPiV + L4, two doses 21 days apart
8	CIRDC (2022)	CPV + CDV + CAV + CPiV + L3, two doses 20 days apart; start age not defined	CPV + CDV + CAV + CPiV + L3 annually	CPV + CDV + CAV + CPiV + L3 Two doses 20 days apart
9	Parvovirosis (2019, unvaccinated litter)	CPV + CDV + CAV + CPiV 8–12–16 weeks + L4: 8–12 weeks Booster dose at 1 year of age CPV + CDV + CAV + CPiV + L4	CPV + CDV + CAV + CPiV Every 3 years + L4 annually	Silent anamnesis: CPV + CDV + CAV + CPiV (MLV) + L4 Two doses 21 days apart Already vaccinated CPV + CDV + CAV + CPiV (MLV) one dose L4: two doses 21 days apart
10	None	CPV + CDV + CAV + CPiV (MLV) + L4: 9–12 weeks	CPV + CDV + CAV + CPiV (MLV) + L4 annually	No defined vaccination protocol

^§^ Not specified = vaccination schedule for that category was not reported in the shelter health management protocol. Abbreviations: CDV, canine distemper virus; CAV, canine adenovirus; CPiV, canine parainfluenza virus; MLV, modified-live vaccine; L2/L3/L4, Leptospira vaccines with different serogroup coverage; L2, *Leptospira interrogans* serogroups Canicola and Icterohemorrhagiae; L3, *Leptospira interrogans* serogroups Canicola, Icterohemorrhagiae, and Grippotyphosa; L4, *Leptospira interrogans* serogroups Canicola, Icterohemorrhagiae, Grippotyphosa, and Autumnalis; CIRDC, canine infectious respiratory disease complex.

**Table 3:** Health management practices and leishmaniasis risk classification in canine shelters.

Shelter	Health Protocol for Newly Admitted Animals ^1^	Risk Class *	Ecto- and Endoparasite Prophylaxis ^§^	Disinfection and Disinfestation
1	Identification; quarantine; clinical exam; testing (*Filaria* and *Leishmania*); ectoparasite prophylaxis; endoparasite treatment after 7–10 days; vaccination after 7 days (booster 21–30 days)	1	Seresto^®^, Frontline^®^, Advantix^®^; Endogard Plus^®^	Bleach; quaternary ammonium compounds; insect traps; mosquito larvicides; environmental insecticides
2	Identification; 10-day quarantine; clinical exam within 72 h; testing (*Filaria* and *Leishmania*); vaccination with booster after 1 month	3	Scalibor^®^, Advantix^®^; Milbemax^®^; Interceptor^®^	Bleach; Lysoform^®^;
3	Identification; clinical exam within 7 days; testing (*Filaria* and *Leishmania*); vaccination; antiparasitic prophylaxis	1	Duecto^®^, Scalibor^®^; Tetrias Plus^®^	Bleach; Lysoform^®^; insect traps
4	Identification; quarantine; antiparasitic prophylaxis; clinical exam within 7 days; testing (*Filaria* and *Leishmania*); vaccination after 10 days	3	Simparica Trio^®^, Scalibor^®^, Advantix^®^; Drontal Plus^®^	Lysoform^®^; insect traps;
5	Identification; 15-day quarantine; clinical exam within 15 days; testing (*Filaria* and *Leishmania*); antiparasitic prophylaxis; vaccination	3	Interceptor^®^, Nemex Pop^®^, Advantix^®^, Scalibor^®^	Bleach; ammonium compounds; Lysoform^®^; insect traps;
6	No clearly defined health protocol for newly admitted animals was available	3	Seresto^®^, Advantix^®^	Bleach; Virkon^®^;
7	Identification; clinical exam within 5 days; testing (*Filaria* and *Leishmania*); antiparasitic prophylaxis; vaccination after 7 days	3	Flevox^®^; Endogard^®^; Pralen^®^	Daily use of bleach; GD90^®^;
8	Identification; clinical exam within 24 h; testing (*Filaria* and *Leishmania*); antiparasitic prophylaxis; vaccination within 24 h and booster after 20 days	1	Advantix^®^; Cardotek^®^	Ammonium compounds; 10-day sanitary vacancy after box emptying; insect traps;
9	Identification; clinical exam within 7 days; testing (*Filaria* and *Leishmania*); antiparasitic prophylaxis; vaccination	3	Seresto^®^, Prevendog^®^; Advantix^®^; Milbemax^®^	RifraxSan^®^; bleach; insect traps;
10	Identification; clinical exam; testing (Giardia, *Filaria* and *Leishmania*); vaccination within 2–3 weeks	3	Seresto^®^, Bravecto^®^; Drontal^®^; Afilaria^®^, Interceptor^®^	Daily use of bleach and Lysoform^®^;

^1^ Further details on vaccination protocols for newly admitted dogs are provided in Table 2. * Shelters were classified according to the Regional Control Plan for Canine Leishmaniasis based on the presence or absence of sandfly vectors and seropositive dogs, resulting in four risk categories. In particular, Category 1 includes shelters where both the vector and seropositive animals are present, indicating a higher risk of *Leishmania infantum* transmission, whereas Category 3 includes shelters located in areas where the vector is present but no seropositive animals have been detected within the facility. ^§^ Commercial product names are reported to reflect actual practices in the shelters and do not imply any endorsement.

## Data Availability

The original contributions presented in this study are included in the article. Further inquiries can be directed to the corresponding author.

## Abbreviations

The following abbreviations are used in this manuscript:

AMR	Antimicrobial resistance
AUSL	Local Health Authority
CIRDC	Canine infectious respiratory disease complex
CPiV	Canine parainfluenza virus
CDV	Canine distemper virus
CPV	Canine parvovirus
CAV	Canine adenovirus
DOI	Duration of immunity
MLV	Modified-live vaccine
PPE	Personal protective equipment
WSAVA	World Small Animal Veterinary Association
